# The Potential of *Pandanus amaryllifolius* Leaves Extract in Fabrication of Dense and Uniform ZnO Microrods

**DOI:** 10.3390/mi11030299

**Published:** 2020-03-13

**Authors:** Rabiatuladawiyah Md Akhir, Siti Zulaikha Umbaidilah, Nurul Afaah Abdullah, Salman A.H. Alrokayan, Haseeb A. Khan, Tetsuo Soga, M. Rusop, Zuraida Khusaimi

**Affiliations:** 1 NANO-SciTech Centre, Institute of Science, Universiti Teknologi MARA, Shah Alam 40450, Malaysia; 2 Faculty of Applied Sciences, Universiti Teknologi MARA, Shah Alam 40450, Malaysia; 3 Research Chair for Biomedical Applications of Nanomaterials, Biochemistry Department, College of Science, King Saud University (KSU), Riyadh 11451, Saudi Arabia; 4 Department of Frontier Materials, Nagoya Institute of Technology (NITech), Nagoya 466-8555, Japan; 5 NANO-Electronic Centre, Faculty of Electrical Engineering, Universiti Teknologi MARA, Shah Alam 40450, Malaysia

**Keywords:** zinc oxide (ZnO), nanorods, microrods, solution immersion method, green synthesis

## Abstract

Zinc oxide (ZnO) micro and nanorods were successfully prepared using *Pandanus amaryllifolius* and hexamethylenetetramine (HMTA) separately as stabilizers using the solution immersion method. Two types of ZnO seed layer were prepared using the same pre-cursor with the different stabilizers. The fabricated ZnO microrods exhibit absorption at ~375 nm as revealed from the UV–Visible absorption spectrum, and this is comparable with ZnO nanorods synthesized using HMTA. X-ray diffraction (XRD) measurement displayed a sharp peak corresponding to the hexagonal wurtzite structure of ZnO microrods. Field emission scanning electron microscopy (FESEM) of ZnO microrods showed average diameter at approximately 500 nm compared to 70 nm of those synthesized from HMTA. A new finding is the ability of *Pandanus amaryllifolius* as a green stabilizer to grow a dense ZnO microrod structure with high crystallinity. Results reveal that both samples from different stabilizers during the preparation of the ZnO seed layer greatly improved the morphological and structural properties and optical absorption of ZnO. The main outcomes from this study will benefit optoelectronic application, such as in ultraviolet (UV) sensors.

## 1. Introduction

Recently, a high yield of zinc oxide (ZnO) nanostructures, using energy-efficient and cost-effective synthesis, has been preferred by various industries such as optoelectronics, photocatalytic, biomedical, agriculture, etc. This demand has led to a progressive synthesis method of ZnO nanostructures. In recent decades, ZnO nanostructures were mainly synthesized using combustion [1,2] and hydrothermal [3,4] methods. However, the synthesis routes of these methods make use of a large number of chemicals and harsh reaction conditions that contribute to environmental contaminants in the form of high energy consumption, heat generation, water consumption, and chemical waste [5,6]. In the following years, sol-gel process became an attractive approach due to its facile and low-cost method [7,8]. The method has been improvised using a sonication-assisted process, which gave advantages towards the elimination of agglomeration of the precursor solution [9]. Recently, the synthesis method has evolved towards a greener approach, such as the solution immersion method, which mostly uses hexamethylenetetramine (HMTA) as a stabilizer. This stabilizer is water-soluble, and the waste can be easily thrown away. However, we noticed that HMTA produced an unpleasant odor and therefore has room for improvement. As an alternative to these conventional chemical and physical methods, there is tremendous progress in the synthesis of nanostructures by using plant-mediated synthesis as reported by the literature.

Silver (Ag) and gold (Au) nanoparticles were among the first to be developed using plant extracts as reductants and stabilizing agents. The metals have been widely investigated for their high anti-microbial activity [10,11,12] and high photocatalytic reactivity [13,14]. Unfortunately, their production involves the use of expensive precursors. Metal oxides such as ZnO, TiO_2_, and SnO_2_ can be an alternative to compete with the materials mentioned above and are suitable candidates due to their lower cost compared to Ag and Au. Among these semiconducting metal oxides, ZnO nanoparticles have received extensive attention due to their versatility in fabrication, which leads to unique morphological and structurally dependent physicochemical properties. In addition to that, ZnO also has an excellent anti-microbial activity [15,16,17] and other applications such as use in photocatalysis [18], gas sensors [19,20], and optoelectronics [21,22]. These biosynthesized ZnO nanoparticles exhibited equivalent properties as compared to those synthesized from the chemical and physical methods.

Recently, highly significant rod-shaped nanoparticles were successfully synthesized using leaves extract, and they possessed superior cellular penetration ability compared to other morphologies [23]. Related to this finding, Vishnukumar [24] reviewed the recent advances and emerging opportunities derived from plant-mediated synthesis for the fabrication of ZnO nanostructures. Almost all parts of plants from barks to seeds were utilized to aid in the synthesis of ZnO in nano forms. From this review, it can be understood that many studies have been conducted and have emphasized ZnO nanoparticle shape. However, ZnO nanostructures can be classified into many types based on their morphology such as nanorods, nanotubes, nanowires, nanoflowers, nanobelts, etc. Among these morphologies, nanorods were extensively studied for their excellent optical and electrical properties [25,26]. In the past few years, ZnO nanorods have risen as the best material for UV sensing usage [27]. This is owing to the superior surface area achieved with nanorods’ diameters and densities [28,29]. However, no extensive studies have been performed in this direction, which dealt with the fabrication of ZnO nanorods by employing the phyto-assisted solution immersion method. Countless plant materials have yet to be explored for the synthesis of ZnO nanorods.

The importance of this study is the enhancement of crystallinity, size uniformity, and rod structure densities of ZnO at low immersion temperature. Using both the solution immersion and phyto-assisted solution immersion method results in a low-cost and environmentally friendly fabrication process. In this study, we report the potential of *Pandanus amaryllifolius* leaves extract as a green stabilizer in the production of ZnO nanostructures. We also compare ZnO nanostructures produced using hexamethylenetetramine (HMTA) as a stabilizer. Even though this green method may not substitute all the existing chemicals used, this is one of the ways to contribute towards lesser damage to the environment. To the best of our knowledge, the structural, morphological, and optical properties of ZnO microrods prepared using *Pandanus amaryllifolius* as a stabilizer via the phyto-assisted solution immersion method, yielding high crystallinity and high microrod density, have not yet been discussed elsewhere. We believe that ZnO nanorods with small diameter size, high nanorod densities, and high crystallinity can be obtained in our future research work and will be beneficial to optoelectronic applications such as UV sensors.

## 2. Materials and Methods

### 2.1. Preparation of Pandanus amaryllifolius Leaves Extract

**Figure 1:**
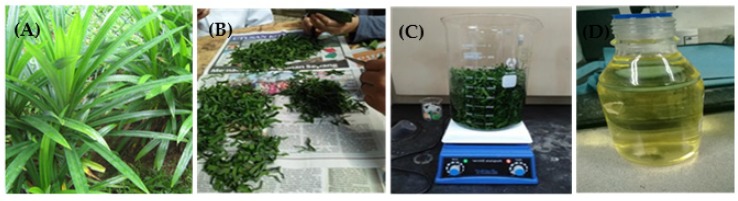
(**A**) *Pandanus amaryllifolius* leaves, (**B**) cutting process, (**C**) boiling process, (**D**) *Pandanus amaryllifolius* leaves extract.

Fresh *Pandanus amaryllifolius* leaves were collected at a local farm and then thoroughly washed. They were then dried under the sun to ensure all remaining moisture was eliminated. The leaves were then finely cut. To prepare the extract for the synthesis of ZnO nanorods, 300 g of the leaves with 900 mL of deionized (DI) water were mixed and boiled at a temperature of 60 °C for 60 min. The extract was cooled to room temperature and was filtered using filter paper before being stored in a refrigerator for further use. Figure 1 shows the preparation of *Pandanus amaryllifolius* extract.

### 2.2. Preparation of ZnO Seed Layers

**Figure 2:**
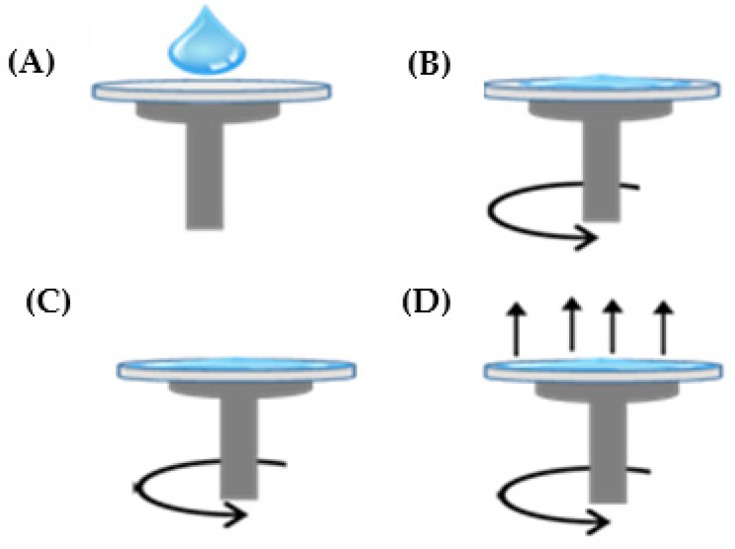
Spin coat process of (**A**) deposition, (**B**) initial spin, (**C**) final spin, and (**D**) evaporation.

The ZnO seed layers were prepared using zinc nitrate hexahydrate (Zn(NO_3_)_2_·6H_2_O),0.4 M) (Sigma-Aldrich, St. Louis, MO, USA) as a precursor dissolved in deionized water with *Pandanus amaryllifolius* leaves extract as a green stabilizer/reduction agent with ratio 1:2 (ZN/Pandan seed layer). A low concentration of 0.05 M sodium hydroxide (NaOH) was added dropwise until reaching pH 9 while stirring. After continuous stirring at 60 °C for 2 h, the sol was aged at room temperature for 24 h. At this point, the precipitate was obtained and later was confirmed as ZnO nanostructures using field emission scanning electron microscopy (FESEM) analysis (FESEM; JEOL JSM 7600F, JEOL Ltd., Akishimi, Japan). On the other hand, the solution prepared was deposited on the glass substrate by the spin-coating method (Laurell Technologies Corporation, North Wales, PA, United States), as shown in Figure 2, at 6000 rpm for 60 s. Ten drops for each layer were then pre-heated for 10 min at 150 °C. This process was repeated five times. After spin-coating the sol on the glass substrate, it was then annealed at 450 °C for 1 h. To compare the effect between *Pandanus amaryllifolius* and HMTA on the growth of ZnO nanostructures, another ZnO seed layer also was prepared by using HMTA (the spin-coating method) as a stabilizer (Zn-HMTA seed layer) and underwent the same synthesis condition as the Zn-Pandan seed layer.

### 2.3. Preparation of ZnO Nanostructures

**Figure 3:**
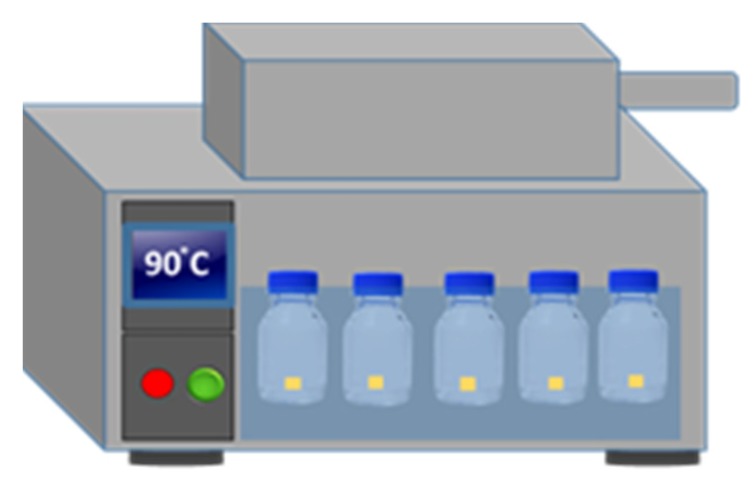
Solution immersion method.

The ZnO nanostructures were grown on both Zn-Pandan and Zn-HMTA seed layers using the solution immersion method (Memmert GmbH + Co. KG, Schwabach, Germany) as shown in Figure 3. The solution was prepared by mixing 0.1 M HMTA and 0.1 M Zn(NO_3_)_2_·6H_2_O. The solution was mixed under continuous stirring at 60 °C for 2 h and was aged at room temperature for 24 h to get a clean and homogenous solution. The resulting solution was poured into a 50 mL centrifuge bottle.

The Zn-Pandan and Zn-HMTA seed layer-coated glass substrates were placed at the bottom of the centrifuge bottle. The centrifuge bottles were sealed and transferred to a water bath for 4 h at 90 °C. After the immersion process, the samples were immediately taken out of the centrifuge bottle. The grown ZnO deposited on the Zn-Pandan and Zn-HMTA seed layer-coated glass substrates was cleaned by rinsing in deionized water and was dried in a furnace at 150 °C for 10 min. For grown ZnO on the Zn-HMTA seed layer, its annealing temperature was controlled at 500 °C for 1 h. This optimum annealing temperature has been established by other researchers especially in the solution immersion method [30,31,32,33,34,35]. ZnO nanorods prepared via the solution immersion method exhibited lower physicochemical properties when annealed at below a temperature of 500 °C. However, no current references have been found on the synthesis of grown ZnO microrods on Zn-Pandan seed layers using the solution immersion method. Hence, ZnO microrods on the Zn-Pandan seed layer were annealed at different temperatures of 100, 200, 300, 400, and 500 °C to identify the optimum structural and optical properties as preliminary data for further morphological characterization.

### 2.4. Characterization

Crystal structure and phases of ZnO were investigated using PANalytical X’Pert Powder X-ray diffractometer (Malvern Panalytical Ltd., Malvern, UK) with a monochromatic Cu Kα (*λ* = 0.154 nm). The crystallite size (*D*) of the ZnO nanostructures was estimated by the Debye–Scherrer formula as in Equation (1):

*D* = K*λ*/(*β*·cos·*θ*) (1)

where:

*D* = the crystal size;
*λ* = the wavelength of the X-ray radiation (λ = 0.15406 nm);
K = usually taken as 0.94;
*β* = the full width at half maximum height;
*θ* = highest diffraction angle.

The optical properties of ZnO nanostructures were investigated by using an ultraviolet-visible model (Cary 60 UV–Vis, Santa Clara, CA, USA). A qualitative preliminary screening test (according to the standard method [36]) was used to examine the presence of secondary metabolic components in the extract of the leaves. To the best of our knowledge, the synthesis of ZnO microrods using *Pandanus amaryllifolius* as a stabilizer was reported for the first time. Hence, a preliminary study of structural and optical properties of ZnO microrods was investigated in order to select the optimum annealing temperature for further characterization. Morphology of ZnO microrods annealed at optimum annealing temperature was then characterized using a field emission scanning electron microscope (FESEM; JEOL JSM 7600F, JEOL Ltd., Akishimi, Japan) with an electron beam energy acceleration voltage of 3 kV.

## 3. Results

### 3.1. X-Ray Diffraction (XRD)

**Figure 4:**
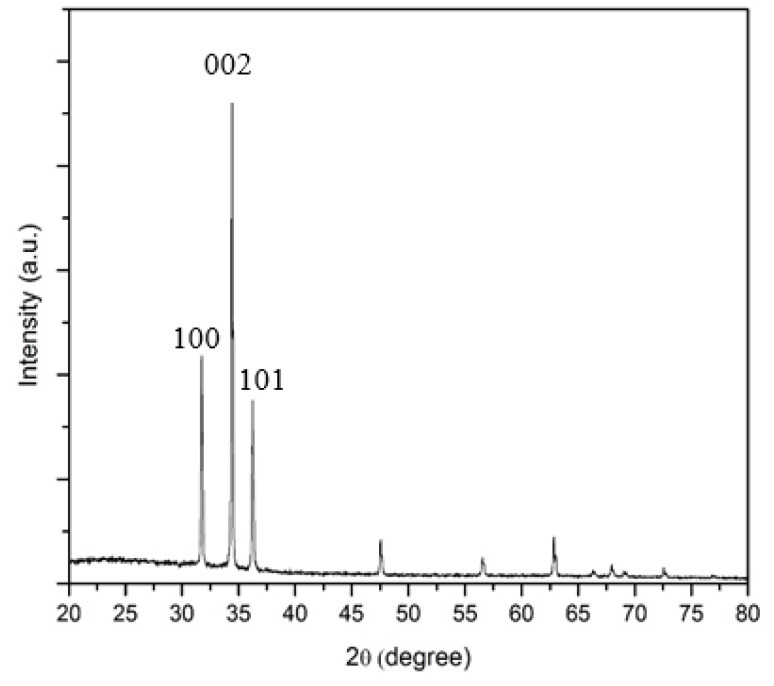
X-ray diffraction (XRD) pattern of ZnO nanorods on Zn-hexamethylenetetramine (HMTA) seed layer.

As a comparison with ZnO nanostructures using Zn-Pandan seed layer, the X-ray diffraction (XRD) pattern of ZnO nanorods synthesized on Zn-HMTA seed layer was first carried out as a reference as shown in Figure 4. The samples exhibit sharp and strong diffraction peaks, which shows that the sample has a high degree of crystallinity. No other peaks were seen, suggesting no impurities other than pure ZnO. It can be indexed as the hexagonal wurtzite structure (ref.code: 01-075-1526). The XRD pattern indicates that mostly three peaks appear at 2*θ* = 32.07, 34.42, and 36.54, which corresponds to the (100), (002), and (101) crystallographic planes of ZnO, respectively, and is in agreement with the result reported from previous literature [37,38].

It was found that the (002) peak is the most dominant, indicating that the nanostructured ZnO grows preferentially along the c-axis (perpendicular to the substrate) and formed nanorods structure. The other (100) and (101) peaks were due to the flower-like ZnO nanorods. It may be attributed to the excess of precursor (Zn(NO_3_)_2_·6H_2_O) concentration (exceeding saturation limit) existing in the sample, leading to excessive growth rate of another ZnO rod on top of grown ZnO nanorods, and hence the formation of flower-like ZnO nanorods. Other than that, crystallographic peaks with weak intensities in the XRD spectra may be due to imperfect nanorod alignment on the substrate [39]. To qualify the preferred orientation of the obtained ZnO films, the relative intensity ratio I_hkl_ is evaluated using Equation (2):

I_hkl_ = I_hkl_/∑I_hkl_ (2)

where:

I_hkl_ = hkl peak intensity;
∑I_hkl_ = sum of the intensities of all the diffraction peaks.

**Figure 5:**
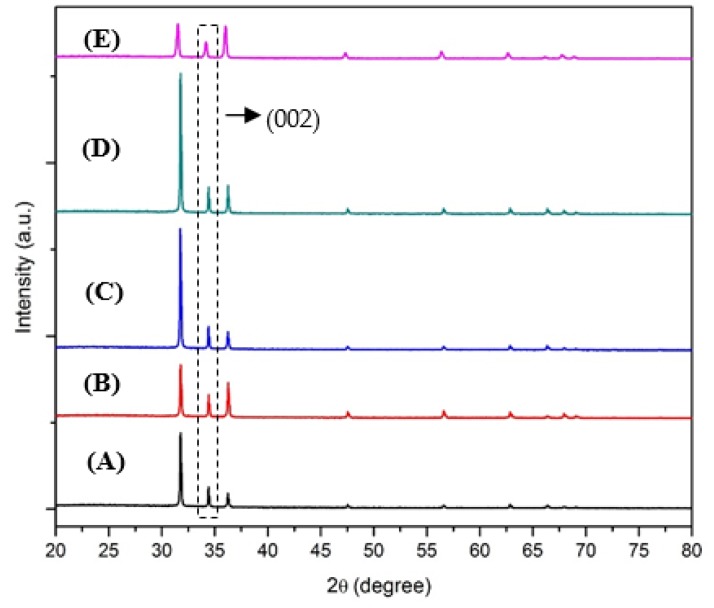
XRD pattern of ZnO microrods on Zn-Pandan seed layer annealed at: (**A**) 100, (**B**) 200, (**C**) 300, (**D**) 400, and (**E**) 500 °C.

The relative intensity ratio of I_(002)_ of ZnO synthesized on Zn-HMTA seed layer is 0.92, which is significantly improved compared to that reported by [40] and [41]. On the other hand, Figure 5 shows the XRD pattern of ZnO nanostructures synthesized on Zn-Pandan seed layer at temperatures of 100, 200, 300, 400, and 500 °C. This sample also has a hexagonal wurtzite structure of ZnO (ref.code: 01-070-2551) and is randomly oriented as compared to the sample in Figure 4. The XRD pattern indicates that mostly three peaks appear at 2*θ* = 31.78, 34.47, and 36.26, which corresponds to the (100), (002), and (101) crystallographic planes of ZnO, respectively.

It was found out that the (100) peak is dominant compared to (002) suggesting the formation of rod-like shape of nanoparticles is dominant compared to highly dense nanorods [36]. Other than that, these results also suggest that by using *Pandanus amaryllifolius* as stabilizer, pure ZnO can also be produced without other impurities. However, to obtain a nanorod morphology that is dominant at peak (002) remains challenging, as observed in Figure 5. The relative intensity of I_(002)_ of ZnO synthesized on the Zn-Pandan seed layer is 0.20 as evident in XRD spectra. This behavior is perhaps linked to the melting point of the phytochemical compounds in *Pandanus amaryllifolius* (flavonoids 273 °C and phenols 40.5 °C). The solution containing mixed phytochemicals with one with a higher melting point and the other one with lower melting point has led to faster evaporation and forces the material to grow along other orientations. Overall, ZnO synthesized at annealing temperature of 400 °C exhibits the highest crystallinity compared to other samples. At 500 °C, the crystallinity of ZnO suddenly decreased as it was beyond the melting point of the biomolecule compound (40 to 273 °C) in *Pandanus amaryllifolius*. The physicochemical properties of Zn-Pandan seed layer annealed at 450 °C were compromised compared to those ZnO seed layers from conventional synthesis. Hence, excessive thermal energy was no longer recrystallized the sample; however, it disrupted the crystallinity. By using the Debye–Scherrer equation, the average crystallite size of ZnO nanostructures synthesized on the Zn-Pandan seed layer is equivalent (11.23 nm) to those sample synthesized from the Zn-HMTA seed layer (12.93 nm).

**Figure 6:**
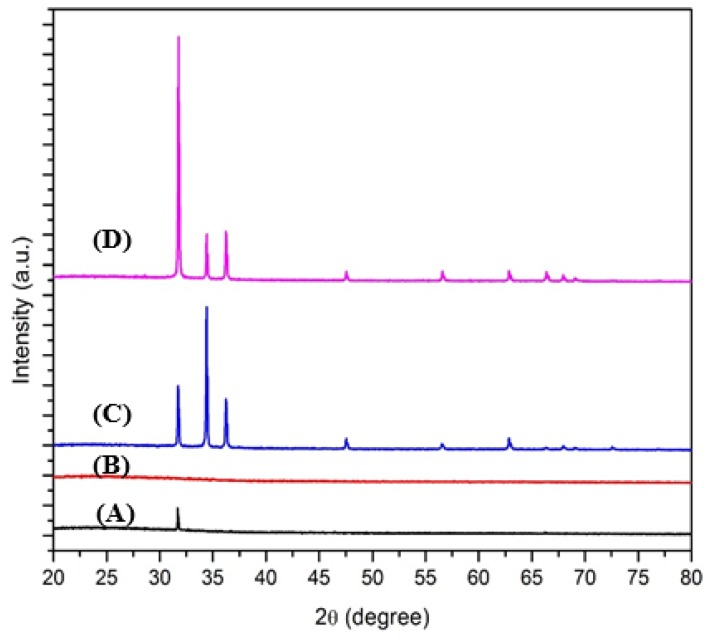
XRD pattern of (**A**) Zn-HMTA seed layer, (**B**) Zn-Pandan seed layer, (**C**) ZnO nanorods on Zn-HMTA seed layer annealed at 500 °C, and (**D**) ZnO microrods on Zn-Pandan seed layer annealed at 400 °C.

Figure 6 shows the XRD pattern of two different seed layers and grown ZnO on respective seed layers annealed at an optimum temperature. The Zn-Pandan seed layer annealed at the same annealing temperature as the Zn-HMTA seed layer (450 °C) was found to have an amorphous structure. The bigger diameter size of grown ZnO on the Zn-Pandan seed layer was affected by the amorphous structure of the Zn-Pandan seed layer. As a comparison with other plant-based stabilizers in the synthesis of rod-shaped ZnO [36], our finding has shown consistent rod shape with a smaller crystallite size with higher crystallinity. As discussed previously in Section 1, higher crystallinity of ZnO is preferred for optoelectronics application, and this could be interesting as we can manipulate natural resources, which are highly abundant towards the synthesis of ZnO micro-nanorods with smaller crystallite size and highly crystalline at comparatively lower production cost. It also should be highlighted that this green alternative approach will benefit the environment as fewer solvents will be thrown away.

Another promising finding is that the crystallinity of ZnO microrods via the phyto-assisted solution immersion method in this study, as shown in Figure 5, was increased approximately six-fold compared to the synthesis of ZnO nanoparticles using *Pandanus amaryllifolius* leaves extract from sol-gel assisted precipitation method in the previous year [42]. This result verifies that plant-based stabilizers in general, and *Pandanus amaryllifolius* in particular, have played a significant in to the growth of ZnO and have potential to be a green alternative reducing and capping agent in the synthesis of highly crystalline ZnO nanostructures through progressive modifications and optimization. The essential ingredients in plant extracts such as terpenoids, alkaloids, phenols, and flavonoids play an important role in stabilizing and reducing and as capping agents in the formation of nanostructures. In various reports, phenols and flavonoids are involved in bio-reduction, stabilization, and formation of metal, metal oxide, and bimetallic nanoparticles. [43,44,45]. From this viewpoint, ZnO nanostructures synthesized from the Zn-Pandan seed layer at 400 °C were chosen to further characterize its morphology, since our aim was to achieve higher intensity of rod shape using the Zn-Pandan seed layer among the different annealing temperatures.

### 3.2. Phytochemical Analysis

Screening analysis for the presence of phytochemical contents in *Pandanus amaryllifolius* was carried out as preliminary data to confirm the role of *Pandanus amaryllifolius* as a stabilizer in the synthesis of ZnO nanorods. In this study, deionized water was used as the solvent for the extraction of plant metabolites, which were allowed to react with zinc nitrate hexahydrate solution. One of the advantages of this method is that mild solvents such as water, ethanol, or methanol also can be used as solvents to react with zinc salt solution under different conditions to obtain maximum yields [46,47,48] In general, plants contain metabolites such as tannins, terpenoids, saponins, starches, polypeptides, flavonoids, and phenols that act both as excellent reducing and capping agents. Screening for presence of flavonoid compounds in *Pandanus amaryllifolius* leaf extract was confirmed by using the Harbone method.

The results of the flavonoid test by using *Pandanus amaryllifolius* leaves extract are tabulated in Table 1. The “+” symbol is used to signify the presence of flavonoid in the leaves extract, which is responsible for the reduction of zinc ions to zinc metal. Pale yellow precipitates, yellow precipitates, and black precipitates were observed in the solution of lead II acetate, sodium hydroxide, and ferric chloride, respectively, after a few minutes. These results were in line with literature over the last year [36]. According to Basnet [49], biosynthesis of ZnO nanostructures is probably driven by the natural phytochemicals (like saponins, polyphenols, terpenoids), which act as both reducing as well as stabilizing (capping) agents. The phytochemicals initiate the reduction of the metal (zinc) to the 0-valence state, and then through calcinations, the oxide may be added to the metal. Another very convincing mechanism is that zinc ions in the solution of the natural extract form complex ions with the polyphenols (or other phytochemicals). This is then followed by the formation of zinc hydroxide (Zn(OH)_2_) through hydrolysis, and finally after calcinations, the complex decomposes, thereby favoring the formation of ZnO nanoparticles [50].

In comparison with the previous methods for the synthesis of ZnO nanostructures, the precipitation method is the easiest and most widely employed. However, highly corrosive NaOH is often used as a reducing agent in the synthesis process. Based on Ul Haq [51], excessive amounts of NaOH with higher molar concentrations are always used to ensure reaction completion. Upon filtration of the reaction mixture, waste is discarded, which might go directly to the wastewater system. This could degrade the metallic parts of the sewage system and also alter the pH of water bodies causing hazards to aquatic flora and fauna. The utilization of *Pandanus amaryllifolius* extract as a reducing agent has minimized the volume of NaOH used in this study. The concentration used was very low (0.05 M) compared to that mostly reported by previous findings [52]. A few studies have attempted biological methods using plant extract as a reducing agent in the synthesis of ZnO nanostructures, especially nanorods. However, the morphology and particle size of ZnO nanorods produced from this phyto-assisted solution immersion method still need to be optimized in the near future.

### 3.3. Ultraviolet-Visible (UV–Vis) Analysis

**Figure 7:**
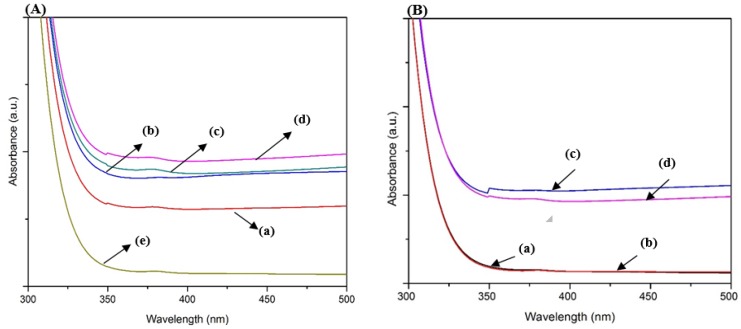
(**A**) UV–Vis absorbance of ZnO microrods on the Zn-Pandan seed layer annealed at temperatures of (**a**) 100, (**b**) 200, (**c**) 300, (**d**) 400, and (**e**) 500 °C. (**B**) UV–Vis absorbance of (**a**) Zn-HMTA seed layer, (**b**) Zn-Pandan seed layer, (**c**) ZnO nanorods on Zn-HMTA seed layer at 500 °C, (**d**) ZnO microrods on Zn-Pandan seed layer at 400 °C.

A UV–Vis spectrometer was used to measure the absorption of the film in the range of 330–800 nm, as depicted in Figure 7. It showed comparatively strong excitonic absorption peaks at 375 nm, which indicate the formation of ZnO. This result is in line with results reported from previous literature [53]. In addition to that, these peaks indicated that the grown ZnO nanorods possessed good optical quality and large exciton binding energy [53]. The results showed that all samples exhibited high absorbance as annealing temperature increased from 100 to 400 °C. This is due to the increase in absorption of photon energy with the increase in annealing temperature.

From this result, it can also be inferred that optical properties of ZnO microrods synthesized on the Zn-Pandan seed layer can be comparatively equivalent to ZnO nanorods synthesized on the Zn-HMTA seed layer. In addition, it is suggested that there was an enhancement in crystallinity of ZnO microrods on the Zn-Pandan seed layer as evident in the XRD pattern in Figure 5. UV–Vis spectra, however, show a dramatic decrease in absorption at an annealing temperature of 500 °C. Excessive thermal energy given to the sample resulted in a higher number of defects formed in the sample annealed at high temperature. The ZnO growth on the Zn-Pandan seed layer lost its crystallinity after being annealed at 500 °C, as evident in Figure 5E. High temperature during the annealing process destructed some of the phytochemicals present in *Pandanus amaryllifolius*. Therefore, lower crystallinity is suspected to be a major cause of lower absorption compared to other samples. Figure 7B shows UV–Vis absorbance of two different seed layers and grown ZnO on respective seed layers annealed at an optimum temperature. It was observed that absorbance of the Zn-Pandan seed layer was similar to the Zn-HMTA seed layer, which holds a potential in new green synthesis. The Zn-Pandan seed layer can be improved in terms of structural and morphological properties to achieve nanosized rod shape for future works. In conclusion, from XRD data and UV–Vis analysis, we chose sample ZnO (Zn-Pandan seed layer) synthesized at an annealing temperature of 400 °C to undergo morphology analysis by FESEM.

### 3.4. Field Emission Scanning Electron Microscopy (FESEM)

**Figure 8:**
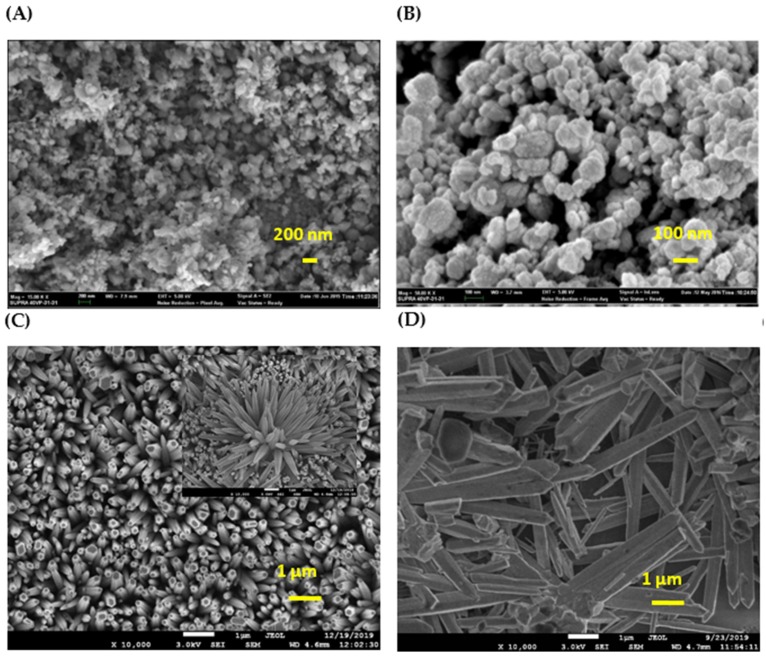
FESEM of (**A**) ZnO nanoparticles using Zn-HMTA, (**B**) ZnO nanoparticles using Zn-Pandan, (**C**) ZnO nanorods using Zn-HMTA seed layer, and (**D**) ZnO microrods using Zn-Pandan seed layer.

Figure 8B confirms that the formation of ZnO nanoparticles was produced using zinc nitrate and *Pandanus amaryllifolius* leaf extract. The results showed an average size of ZnO at 24.15 nm, slightly smaller than that synthesized from HMTA, 30.54 nm, as shown in Figure 8A. ZnO nanoparticles using *Pandanus amaryllifolius* as stabilizer exhibited similar nanostructure compared to using HMTA. This finding could be promising for manipulation of the phytochemical compound in *Pandanus amaryllifolius* in the reduction of zinc nitrate into zinc oxide and could help the stabilization process throughout the synthesis. Here, we combined the advantages of two types of nanostructures from the solution immersion method as shown in Figure 8. ZnO nanoparticles are favored for applications such as antimicrobial, corrosion, and photocatalytic application. On the other hand, ZnO nanorods have always been preferred for optoelectronic and gas sensor applications, as discussed in Section 1. As evident in Figure 8C, a dense and high uniformity of ZnO nanorods was produced using the Zn-HMTA seed layer. Xu Yang [54] also used the ZnO seed layer for the formation of ZnO nanorods by a hydrothermal method. They found out that the bare ZnO nanorods are approximately in the shape of hexagonal prisms with their axes almost perpendicular to the substrate and having an average diameter of 90 nm. Interestingly in our findings, through this simple and cost-effective solution immersion method, the hexagonal diameter becomes smaller with an average diameter of 71.67 nm. In addition, the shape of the nanorods is denser and exhibits uniformity in size and shape. Inset of Figure 8C showes the flower-like shape of ZnO nanorods produced as evident from (100) and (101) peak in XRD pattern.

We also synthesized ZnO microrods using the Zn-Pandan seed layer to see the potential of *Pandanus amaryllifolius* as reducing/stabilizing agent in the synthesis of nanostructures. It is observed that when ZnO is grown on the Zn-Pandan seed layer, the microrods are less hexagonal, and the diameter is increased to approximately 500 nm. The solid structure of ZnO was formed from tightly bonded nanorods [55]. As observed in Figure 8D, even though the diameter size of ZnO microrods is bigger than that of ZnO nanorods in Figure 8C, the shape and size uniformity are significant. The Zn-Pandan seed layer is still able to produce ZnO without impurities and with dense microrod structures. The Zn-Pandan seed layer may not be conducive to growing smaller, more uniform, and denser ZnO nanorods due to the high annealing temperature of 450 °C of the Zn-Pandan seed layer. This high energy force may degrade the functional groups that are responsible for stabilizing the zinc metal into monodispersed nanorods. From XRD, UV–Vis, and FESEM analysis, it was understood that ZnO nanorods synthesized from Zn-HMTA seed layer possessed better physicochemical properties compared to those synthesized from the Zn-Pandan seed layer. However, we also managed to highlight that this solution immersion method have potential to be easily tailored using green stabilizers with different annealing temperatures, which leads to a dense microrod structure and enhanced crystallinity compared to ZnO in nanoparticle shape. For future study, Zn-Pandan seed layer will be studied with emphasis given to the preparation of raw material (sun-dried, commercial-dried, and freeze-dried *Pandanus amaryllifolius*) to fine-tune optimum parameters to obtain ZnO rods in nano size.

## 4. Conclusions

In summary, we have successfully synthesized ZnO microrods on the ZnO seed layer using *Pandanus amaryllifolius* extract as a stabilizer and have compared with ZnO nanorods synthesized on the ZnO seed layer using HMTA as a stabilizer. The advantages of producing two different nanostructures through the phyto-assisted solution immersion method are reported for the first time. The (002) peak for ZnO microrods on Zn-Pandan seed layer and on Zn-HMTA seed layer were clearly confirmed by XRD. Both samples showed a hexagonal wurtzite crystal structure with high purity. Both ZnOs were further confirmed by UV–Vis at *λ*_max_ of 373 nm. It was challenging to replicate exactly the same structural, optical, and morphological properties as obtained from ZnO nanorods on Zn-HMTA. However, the growth of ZnO microrods on the Zn-Pandan seed layer is expected to achieve new changes in the field of UV sensors due to its dense and highly uniform microrod structures and high crystallinity using a local native plant (*Pandanus amaryllifolius*) as a cheaper and environmentally friendly stabilizer. These findings bridge the gap between conventional biosynthesis and the phyto-assisted solution immersion method and can be improved through a continuous parameter optimization process in the near future. Overall, the advantages of low working temperature, cheap raw materials, and most importantly, the least possible damage to the environment have added value to these findings.

## Figures and Tables

**Table 1:** Flavonoids test for *Pandanus amaryllifolius* leaves extract.

Test	Observation	Result
Lead acetate	Pale yellow precipitate	+
Sodium hydroxide	Deep yellow precipitate	+
Ferric chloride	Black precipitate	+

“+” symbol is used to signify the presence of flavonoid in the leaves extract.
